# The Relationship between Sport Participation and Chronic Diseases among Men in the USA: An Examination of the Behavioral Risk Factor Surveillance System

**DOI:** 10.3390/sports5030056

**Published:** 2017-08-02

**Authors:** Jennifer Pharr, Nancy Lough

**Affiliations:** 1 School of Community Health Sciences, University of Nevada, Las Vegas, NV 89154, USA; 2 College of Education, University of Nevada, Las Vegas, NV 89154, USA; nancy.lough@unlv.edu

**Keywords:** physical activity, sport participation, conditioning exercise, chronic diseases, United States

## Abstract

Sport participation has been associated with lower rates of chronic diseases when compared to other forms of physical activity (PA) among women. However, we do not know if this relationship holds true for men. The purpose of this study was to examine the relationship between sport participation and men’s health and chronic diseases in the USA. This study was a secondary data analysis of the 2015 national Behavioral Risk Factor Surveillance System (BRFSS) survey. Participants were questioned extensively about their PA behaviors. Seventy-six different activities were identified and these activities were categorized as sport, conditioning exercise, recreation, or household tasks based upon previously identified categories. Logistic regression was utilized to calculate odds and adjusted odds ratios for chronic diseases based on physical activity category. When compared to men who participated in sport, men in the other PA categories had significantly higher odds for all of the chronic diseases except asthma. After controlling for demographic variables, significant odds remain except for stroke. Higher odds for chronic diseases in the other PA categories indicates that men in these group have a higher risk for chronic diseases than men in the sport category. Because of the potential health improvements related to sports participation, it is important to maintain and increase sport participation for both adolescents and adults.

## 1. Introduction

Numerous health benefits are associated with physical activity (PA) [1,2,3,4,5]. These include a reduced risk of heart disease and risk factors for heart disease (high blood pressure, high cholesterol, and diabetes), and reduced risk of some forms of cancer [1,2,3,6,7]. Additionally, being physically active has been found to improve bone density, pulmonary function, mood and wellbeing [1,2,3,6,7,8,9,10]. Most PA research has compared people who are physically active to those who are not and have grouped all types of PA together. However, some research has examined the health benefits based on the intensity of PA as measured by metabolic equivalents (METs). METs is a physiological measurement of the energy cost of PA based on calories per kilogram of body weight per hour (kcal/kg/h). One METs is the energy cost of resting. The METs value of different forms of PA compare the intensity of that PA to rest. For example, the METs value of playing basketball is 11.1, so playing basketball is 11.1 time more intense than resting. Research finds that people who engage in vigorous (METs value of 6 or greater) PA have better health outcomes compared to people who perform less vigorous PA [11,12,13,14,15].

PA can occur in multiple forms and can be separated into categories including sport, conditioning exercise, recreation, and household tasks [16,17]. Many sports have a METs value greater than 6 and are considered to be vigorous activity [18]. For example, the METs value assigned with basketball is 11.1 and with soccer is 10.3 [18]. This leads us to hypothesize that people who achieve a higher METs value through sports participation would have better health outcomes when compared to people who participate in other forms of PA that do not achieve a high METs level.

Research examining the health benefits of sport participation for adolescents has found that adolescents who participate in sport are more vigorously active, less likely to be overweight or engage in risky health behaviors, and more likely to participate in sport or other forms of PA as adults [19,20,21,22,23]. However, few studies have examined the health impact of sport specifically for adults. In our study of sport participation and women, we found that women who participated in sport did so at a higher METs value (average METs = 6.18) and were less likely to report several chronic diseases and conditions including heart attack, cardiovascular, high blood pressure, high cholesterol, asthma, other cancers, chronic obstructive pulmonary disease (COPD), arthritis, depression, kidney disease, and diabetes when compared to women who participated in the other categories of PA (conditioning exercise, recreation, or household tasks) [16]. Oja and colleagues found that specific sports participation (cycling, swimming and racquet sports) reduced all-cause mortality [24]. Other studies focused on the psychological benefits of sport participation have found that adults who participate in sport have higher levels of happiness, well-being, confidence, mental health and vitality, and lower levels of stress when compared to adults who participate in other forms of physical activity [25,26,27,28,29,30]. However, we do not know about the relationship between sport participation and chronic diseases in men. 

To add to this growing field of research and to further the understanding of the health impact of sport, the purpose of this study was to examine the relationship between sport participation and men’s health in the USA. We wanted to see if men who participate in sport reported fewer chronic conditions than men who participated in other forms of PA. Additionally, we intended to determine if men who participated in sport did so at higher METs level than men who participated in other forms of PA and if they were more likely to meet the recommended amount of PA per week. Our hypotheses guiding the study were:

1. Men who report participating in sport will be significantly less likely to report chronic diseases than men who report participating in conditioning exercise, household tasks, or recreation, and differences will remain after adjusting for demographic characteristics including: age, income, marital status, education, and race/ethnicity.
2. Men who report sport participation will be significantly more likely to meet the recommended amount of exercise per week and achieve a higher METs value compared to men who participate in conditioning exercise, household tasks, or recreation.

## 2. Materials and Methods

We employed a similar methodology to our study of the health benefits of sport for women in the USA which utilized 2013 data [16].

### 2.1. Study Design and Setting

This study was a cross-sectional, secondary analysis of 2015 Behavioral Risk Factor Surveillance System (BRFSS) data. The BRFSS is the largest health-related survey of adults in the United States of America (USA). The BRFSS started in 1984, is conducted annually and is a collaboration between the Centers for Disease Control and Prevention (CDC) and USA states and territories. 

### 2.2. Participants

The BRFSS is a random-digit dial telephone (cellular and landline telephones) survey which targets non-institutionalized adults 18 years of age and older [31]. Participants from all USA states and territories are included in the survey. In 2015, 441,456 people participated in the BRFSS. Disproportionate stratified sampling is employed to provide an adequate sample size for smaller demographic areas [31]. Data are weighted for population attributes and non-response [31]. Detailed information about the BRFSS weighting, sampling, and survey administration can be found at https://www.cdc.gov/brfss. 

### 2.3. BRFSS Survey and Variables

The core component of the BRFSS survey includes questions that are asked of all participants and includes questions about their demographics, preventive health practices, chronic diseases, and health risk behaviors. In odd years, participants are questioned extensively about their exercise behaviors [32]. The initial exercise question is, “During the past month, other than your regular job, did you participate in any physical activities such as running, calisthenics, golf, gardening or walking for exercise?” [32]. Participants who answered “yes” to this question are then asked more specific questions about their exercise. The next question is, “What type of physical activity or exercise did you spend the most time doing the past month?” [32]. This is an open-ended question, and participants can only identify one activity or exercise for this question. Seventy-six different activities have been reported by the participants. Next the participants are asked, “How many times per week or per month did you take part in this activity during the past month?” followed by, “And when you took part in this activity, for how many minutes or hours did you usually keep at it?” [32]. 

Based on the responses to those questions an algorithm is employed to classify respondents as to whether or not they met aerobic exercise recommendations. The recommended amount of aerobic exercise is defined by the CDC for the BRFSS as: “Meeting aerobic recommendations”—respondents who report doing 150+ min (or vigorous equivalent) of aerobic exercise or “Not meeting aerobic recommendations”–respondents who report doing insufficient PA (0–149 min of aerobic exercise) [32]. Additionally, based on the answer to the exercise question, the CDC assigns a METs value to the activity. 

Constructed from the answers to the exercise questions (activity, duration, frequency), the CDC calculates variables for each participant regarding their PA category (highly active, active, insufficiently active, or inactive) and whether they met the recommended amount of aerobic exercise or not. PA levels set by the CDC for the BRFSS are determined as follows: *Highly Active*—respondents who reported doing enough PA to meet the 300 min of aerobic activity or 150 min of vigorous aerobic exercise; *Active*—respondents who reported doing 150–300 min of aerobic activity (or the vigorous equivalent); Insufficiently *Active*—respondents who reported doing insufficient PA (11–149 min of aerobic activity); *Inactive—*respondents who reported doing no PA [32]. The BRFSS has been found to have high reliability (test/retest comparisons) and validity (compared with other surveys, participant logs, accelerometers, or other PA measures) for the PA questions especially for those who report high levels of PA [33].

Participants provide demographic data including sex, age, employment, education, race/ethnicity, income, and marital status. They are asked about chronic conditions including: heart attack, cardiovascular disease (CVD), stroke, high blood pressure, high cholesterol, asthma, skin cancer, other cancers, chronic obstructive pulmonary disease (COPD), arthritis, depression, kidney disease, diabetes and overweight/obesity. 

For this study, men who answered “no” to the initial exercise question (During the past month, other than your regular job, did you participate in any physical activities such as running, calisthenics, golf, gardening or walking for exercise?), who refused to answer the second exercise question (What type of physical activity or exercise did you spend the most time doing the past month?), and women were excluded from our analysis. Answers to chronic disease questions were dichotomized as “yes” or “no” with “refused to answer” or “I don’t know” considered missing.

Two researchers, one with a background in sport and one with a background in PA, had previously reviewed the 76 different activities and independently placed them into 4 predetermined leisure time PA categories of: sport, conditioning exercise, household tasks, and recreation (Table 1) [16]. The 4 categories were a modification of the 4 categories provided and described by Caspersen et al., which included: sport, conditioning exercise, household tasks, and other [17]. We used Caspersen et al.’s descriptions as well as definition of sport provided above to categorize the different activities and to differentiate sport from the other forms of activity [17]. We agreed on the categories 96% of the time. The 3 activities that we did not agree upon were discussed and we were ultimately able to agree on their categorization [16]. Participants could only indicate one activity or exercise for the question and could not be counted in multiple categories. 

### 2.4. Statistical Analyses

SAS version 9.2 (SAS Institute Inc., Cary, NC, USA) was used for statistical analyses of demographic characteristics and chronic conditions/health risk behaviors by exercise category. Weighted descriptive statistics were performed to describe the demographic characteristics of the 4 exercise categories by age, race, education, income, employment, and marital status. To determine statistically significant differences in demographic characteristics, PA level and aerobic recommendation achieved by exercise category, Rao X square tests were performed using PROC SURVEYFREQ in SAS. When the overall Rao X square tests were significant, indicating that there was a significant difference between at least two groups, we performed post hoc paired comparisons to determine if those who participated in sport were significantly different than those who participated in the other exercise categories. To reduce type I error, we performed a Bonferroni correction to the p-value for each demographic variable by dividing the original *p*-value (alpha_original_ = 0.05) by the number of comparisons made for the given variable. The Bonferroni corrected *p*-values were: marital status *p* = 0.0014, educational attainment *p* = 0.0014, age *p* = 0.0012, race/ethnicity *p* = 0.0017, income *p* = 0.0017, and employment *p* = 0.0021. Additionally, PROC SURVEYMEANS in SAS was used to calculate the mean number of minutes and the mean METs associated with the activity for each of the PA categories along with a 95% confidence interval (CI) to compare groups. If the 95% CI’s did not overlap, then the groups were significantly different. Logistic regression was used to calculate crude and adjusted odds ratios for chronic conditions and risk factors by exercise category with Sport as the reference category. Because there were significant differences in overall Rao X square tests for all of the demographic variables, we used multiple logistic regression to control for all demographic variables when calculating adjusted odds ratios. Logistic and multiple logistic regression were warranted for these calculations because the dependent variables were dichotomous in that either the participant reported the chronic disease or risk factor (yes) or did not report the chronic disease or risk factor (no). 

This study was deemed as excluded by the University of Nevada, Las Vegas Institutional Review Board as it was a secondary data analysis of de-identified data.

## 3. Results

### 3.1. Demographic Characteristics

Of the 441,456 participants, 186,938 were men. Of the men, 118,665 reported participating in some form of PA with 18.8% participating in sport, 68.5% participating in conditioning exercise, 6% participating in recreation, and 7% participating in household tasks (Table 2). The overall Rao chi square tests were significant for each demographic variable indicating that there was a significant difference between at least two groups for all demographic variables. Post hoc analyses comparing men who participated in sport to men who participated in each of the other exercise categories were all significant with a *p-*value < 0.001 indicating that men who participated in sport were significantly different than men who participated in each of the other exercise categories based on their demographic characteristics. A higher percentage of men participating in sport reported being single; a college graduate; Hispanic; in the 18–24, 25–34 and 35–44 age groups; employed; and making more than $75,000 when compared to the percentage reported by men in the other PA categories (Table 2). 

### 3.2. Physical Activity Time and Intensity and General Health

Compared to men who participated in other categories of PA, a higher percentage of men who participated in sport reported excellent-good general health; however, a lower percentage of them met the recommendations for PA compared to men who participated in recreation or household tasks (Table 3). Additionally, a lower percentage of men who participated in sport were rated as highly active when compared to men who participated in recreation. When considering time and intensity (METs), men who participated in sport did so at a significantly higher average METs level than men in the other PA categories; however, men who participated in sport spent a significantly shorter amount of time compared to men who participated in recreation and household tasks (Table 4). 

### 3.3. Odds and Adjusted Odds Ratios for Chronic Diseases

Results for odds ratios and adjusted odds ratios are presented in Table 5 and Table 6. Each PA category is compared to sport. When compared to men who participated in sport, men in the other PA categories had significantly higher odds ratios for all of the chronic conditions except asthma (Table 5). Men who participated in conditioning exercise, recreation, or household tasks where significantly more likely to report having: high blood pressure, high cholesterol, heart attack, cardiovascular disease, stroke, skin cancer, other cancers, chronic obstructive pulmonary disease, arthritis, depression, and diabetes. Additionally, men who participated in conditioning exercise, recreation, or household tasks were significant more likely to be overweight/obese and to smoke than men who participated in sport; however, men who participated in sport were significantly more likely to binge drink than men who participated in conditioning exercise or household tasks (Table 5). 

After controlling for demographic variables (adjusted odds ratios), almost all of the significant odds ratios remain except there was no longer a significant difference (1) between men who participated in conditioning exercise, recreation, or household tasks and men who participate in sport for stroke; (2) between men who participated in household tasks and men who participated in sport for skin cancer; (3) between men who participated in recreation and men who participated in sport for other types of cancer (Table 5). Asthma became significant for the men who participated in conditioning exercise and household tasks compared to men who participated in sport (Table 6). Additionally, there was no longer a significant difference between men who participated in conditioning exercise or household task and men who participated in sport for binge drinking; however, men who participated in recreation were more likely to binge drink than men who participated in sport. 

## 4. Discussion

We found that men who participate in sport were more likely to do so at a higher METs level and were less likely to report chronic diseases or conditions. This finding is consistent with our previous research on the health impact of sport for women and serves as a contribution to other studies addressing the health impact of sport among adults and adolescents [19,20,21,22,23,24,25,26]. Sport may be a viable mechanism to help adults achieve exercise intensities which are conducive to improve health. Our finding supports a study by Tanasescu and colleague, who observed greater health benefits and reduced risk among people who exercised at a greater intensity (vigorously) when compared to a lower intensity (moderate or low) [34]. While the mechanisms to explain the association between exercise and improved health are numerous, the added health benefit of vigorous exercise may be due to higher aerobic fitness and better energy balance [34]. This study adds to the growing body of literature examining the impact of sports participation on health.

Sport is beginning to be recognized for its potential to improve the health of both adolescents and adults [16,25,26,27,28,29,30,35]. For adolescents, sports participation may be doubly impactful because adolescents who participate in sports are more likely to continue participation as adults [36]. However, in the United States, there has been a consistent decrease in sports participation among youth and adolescents [37]. Research from the Aspen Institute saw a 10.7% decrease in soccer participation, a 28.6% decrease in football participation, and a 31.3% decrease in softball participation between 2008 and 2013 among 6–12 year olds in the USA [37]. This means that 2.6 million fewer children were participating in sports over a five year time period. This decrease may have both short and long-term health implications as these children are not reaping the health benefits now because they are not participating in sports, and they most likely will not participate in sport as adults, thus missing out on potential health benefits in adulthood.

We also found that sports participation decreases with increasing age. This supports the findings from other studies in the United State and Australia [36,38]. A 2015 Robert Wood Johnson report shows that although 75% of adults reported having participated in sport in their youth, only 25% reported currently playing sports [36]. They also found a decrease in participation with age as 41% of people 22 to 25 years old played sports compared to 20% of those older than 50. Of the adults who no longer participated in sports, health related issues, lack of time, and lack of interest were the many reasons they stopped. Adults who continue to participate do so for personal enjoyment and to improve their health [36].

Because of the potential health improvements related to sports participation, it is important to maintain and increase sport participation for both adolescents and adults. Many European countries have developed “sports for all” policies to improve sports participation and health among their populous; however, these policies do not always enjoy the same governmental support as their national institutes for elite sport [38]. This prioritization of elite sport over mass participation has not resulted in high rates of sports participation, as is the case when mass participation is prioritized [39]. However, when priority is given to increase mass participation in sport, more people participate. For example, Ireland’s Building pathways in Irish Sport provides recreational pathways to ensure lifelong involvement in sport [40]. Additionally, Finland and the Netherlands also invest more money and political emphasis on mass participation sport, and as a result have higher participation rates than many European countries [39]. Increasing sports participation within the population requires the engagement of government, the sport industry, community, and professional sports organizations.

As with any study, there are limitations to this research. Because the BRFSS is cross-sectional, causation cannot be determined [41]. Also, because the data collected in the BRFSS are self-reported, there is the possibility of self-report bias. Participants may have under or over reported based on their perception of social acceptability [42]. 

## 5. Conclusions

Sport participation was associated with more vigorous PA and lower odds of chronic diseases among men in the USA. A previous study has found the same results among women in the USA. However, sport participation declines in adulthood among both genders. Countries that have prioritized mass participation sport, both financially and politically, have seen more people participating in sport. These initiatives may serve as examples to guide efforts in the US to increase adult sport participation. Because sport participation is associated with better health outcomes, even though participants spent less time engaged in sport as compared to other exercise categories, sport appears to be a more effective model for improved health. 

## Figures and Tables

**Table 1:** Exercise categories for reported activities.

Sport	Conditioning Exercise	Recreation	Household Tasks
Badminton	Active Game Device (i.e., Wii)	Backpacking	Carpentry
Basketball	Aerobics class	Boating	Childcare
Bicycling	Bicycle machine	Bowling	Farming/ranching
Boxing	Calisthenics	Canoeing	Gardening
Golf	Dancing	Fishing	Housework (vacuuming)
Handball	Elliptical machine	Frisbee	Mowing lawn
Hockey	Inline skating	Hiking	Painting house
Lacrosse	Jogging	Horseback riding	Raking lawn
Mountain climbing	Karate	Hunting—small and large game	Snow blowing
Racquetball	Pilates	Paddleball	Snow shoveling
Running	Rope skipping	Snorkeling	Yard work
Ruby	Rowing machine	Stream fishing	
Rock climbing	Scuba diving	Swimming—not laps	
Soccer	Skateboarding	Table tennis	
Softball/baseball	Skating—ice	Waterskiing	
Squash	Snow skiing		
Tennis	Snowshoeing		
Touch football	Stairmaster		
Volleyball	Surfing		
Wrestling	Swimming—laps		
	Tai chi		
	Walking		
	Weight lifting		
	Upper body cycle		

**Table 2:** Demographic Characteristics by Exercise Type–Percentages and Overall Rao X**^2^**.

Demographic variables	Total	Sport	CE	Recreation	HT	Overall X^2^ & *p-*Value
**N(%)**	118,665	22,266 (18.8)	81,224 (68.5)	6938 (6%)	8237 (7%)	
**Marital Status**	%	%	%	%	%	1345, *p <* 0.01
Married	70,576 (59.5)	53.5	59.8	64.1	68.2	
Divorced	13,236 (11.2)	8.3	12.0	11.1	10.9	
Widowed	6499 (5.5)	1.7	6.4	5.6	6.9	
Separated	1858 (1.6)	1.4	1.7	1.3	1.2	
Single	22,455 (18.9)	30.5	17.0	14.7	9.8	
Partnered	3751 (3.2)	4.2	2.9	3.1	2.9	
**Educational Attainment**						127, *p <* 0.01
College graduate	52,528 (44.3)	49.8	43.6	45.1	35.5	
Did not graduate HS	6853 (5.8)	4.8	6.1	3.2	7.1	
High school graduate	29,031 (24.5)	20.8	24.9	24.2	29.9	
Some college	30,253 (25.5)	24.6	25.4	27.5	27.5	
**Age**						3926, *p <* 0.01
18–24	8803 (7.4)	18.2	5.3	5.2	1.3	
25–34	13,110 (11.0)	20.7	9.0	10.4	5.4	
35–44	14,360 (12.1)	18.4	10.8	11.8	8.3	
45–54	19,377 (16.3)	16.3	16.4	15.8	16.0	
55–64	25,827 (21.8)	14.1	23.6	20.4	25.9	
64–74	23,366 (19.7)	8.4	21.8	23.5	26.6	
75+	8803 (7.4)	18.2	5.3	5.2	1.3	
**Race/Ethnicity**						455, *p <* 0.01
White	92,735 (78.1)	72.0	78.4	87.2	84.8	
Black	7279(6.1)	6.6	6.6	2.3	3.3	
Other	5822 (4.9)	6.7	4.7	3.8	3.4	
Multi	2367 (2.0)	2.2	2.0	1.8	1.8	
Hispanic	8725 (7.4)	11.2	6.9	3.6	5.1	
**Income**						229, *p <* 0.01
>75 K	42,980 (36.2)	43.0	34.7	40.7	29.2	
<10 K	3363 (2.8)	2.5	3.1	1.1	2.6	
10–25 K	15,810 (13.3)	10.7	14.3	8.7	14.1	
25–50 K	23,947 (20.2)	17.3	20.5	20.1	25.2	
50–75 K	18,160 (15.3)	14.4	15.2	17.8	16.8	
**Employment**						744, *p <* 0.01
Employed	69,202 (58.3)	74.4	54.7	59.7	49.4	
Unemployed	5195 (4.4)	4.5	4.5	2.8	4.4	
OLF	38,406 (32.4)	19.4	34.8	35.3	41.3	
Unable to work	5466 (4.6)	1.2	5.7	1.9	4.8	

Conditioning exercise = CE; Household tasks = HT; OLF = out of labor force.

**Table 3:** General Health and Exercise Amounts by Exercise Type.

Variable	Sport (%)	CE (%)	Recreation (%)	HT (%)	X^2^ & *p*-Value
**General Health**					390, *p* < 0.01
Excellent/very good, good	94.9	85.0	89.5	82.6	
Fair/poor	5.3	15.1	10.6	17.4	
**Physical Activity Level**					1067, *p* < 0.01
Highly active	51.5	40.5	67.5	46.0	
Active	24.7	24.6	17.4	23.5	
Insufficiently active	20.2	27.4	10.4	24.0	
Inactive	0.7	2.4	1.5	2.0	
**Aerobic Exercise Recommendations**					584, *p* < 0.01
Met aerobic recommendations	76.8	65.9	85.6	82.8	
Did not meet aerobic recommendations	20.9	29.9	11.9	13.5	

Conditioning exercise = CE; Household tasks = HT.

**Table 4:** Exercise Minutes and Metabolic Equivalence (METs) by Exercise Type.

Variable	Sport Mean (95% CI)	CE Mean (95% CI)	Recreation Mean (95% CI)	HT Mean (95% CI)
Minutes of Exercise	243.47 (236.21–250.74)	209.86 (200.74–218.97)	357.30 (340.69–373.90)	451.06 (425.57–476.55)
METs	62.93 (62.70–63.15)	34.00 (33.72–34.23)	47.30 (46.74–47.87)	48.56 (48.30–48.83)

Conditioning exercise = CE; Household tasks = HT; CI = Confidence Interval.

**Table 5:** Odds Ratios for Chronic Conditions and Risk Factors with Sport as Reference.

Chronic Condition	Conditioning Exercise		Recreation		Household Tasks	
	OR	95% CI	OR	95% CI	OR	95% CI
High Blood Pressure	2.73 *	2.56–2.91	2.39 *	2.15–2.68	3.52 *	3.18–3.89
High Cholesterol	2.04 *	1.91–2.18	2.01 *	1.79–2.27	2.45 *	2.20–2.73
Heart Attack	5.16 *	4.25–6.26	4.69 *	3.44–6.40	5.89 *	4.66–7.45
CVD	5.45 *	4.38–6.77	4.92 *	3.78–6.41	5.85 *	4.52–7.57
Stroke	3.86 *	2.73–5.47	2.63 *	1.73–3.99	5.02 *	3.38–7.45
Asthma	0.98	0.90–1.07	0.86	0.73–1.01	0.86	0.74–0.99
Skin Cancer	2.94 *	2.64–3.28	4.35 *	3.67–5.17	4.44 *	3.82–5.17
Other Cancers	3.54 *	3.08–4.08	3.47 *	2.86–4.22	5.10 *	4.24–6.14
COPD	2.75 *	2.30–3.31	2.77 *	2.09–3.68	3.99 *	3.19–5.00
Arthritis	3.27 *	3.00–3.55	3.55 *	3.13–4.03	5.07 *	4.52–5.70
Depression	1.71 *	1.56–1.88	1.46 *	1.25–1.71	1.71 *	1.49–1.98
Kidney Disease	2.92 *	2.17–3.94	2.66 *	1.81–3.91	3.69 *	2.56–5.34
Overweight/obese	1.84 *	1.74–1.95	1.95 *	1.74–2.18	2.00 *	1.79–2.22
Diabetes	4.80 *	4.14–5.55	3.89 *	3.19–4.75	4.81 *	4.03–5.74
Current Smoker	1.25 *	1.04–1.22	1.49 *	1.30–1.69	1.70 *	1.51–1.94
Binge Drinking	0.65 *	0.61–0.69	0.95	0.85–1.07	0.65 *	0.58–0.73

* Significant Odds Ratios.

**Table 6:** Adjusted Odds Ratios for Chronic Conditions and Risk Factors with Sport as Reference.

Chronic Condition	Conditioning Exercise		Recreation		Household Tasks	
	AOR	95% CI	AOR	95% CI	AOR	95% CI
High Blood Pressure	1.42 *	1.32–1.53	1.24 *	1.10–1.40	1.43 *	1.28–1.60
High Cholesterol	1.25 *	1.15–1.35	1.18 *	1.04–1.35	1.23 *	1.08–1.41
Heart Attack	1.76 *	1.41–2.19	1.55 *	1.17–2.04	1.39 *	1.07–1.83
CVD	1.88 *	1.50–2.36	1.68 *	1.27–2.21	1.35 *	1.02–1.78
Stroke	1.32	0.88–1.98	1.07	0.68–1.70	1.41	0.90–2.19
Asthma	1.15 *	1.04–1.27	1.08	0.90–1.30	1.21 *	1.02–1.43
Skin Cancer	1.13 *	1.00–1.29	1.41 *	1.15–1.72	1.13	0.95–1.34
Other Cancers	1.41 *	1.20–1.65	1.23	0.98–1.54	1.56 *	1.27–1.91
COPD	1.38 *	1.12–1.71	1.55 *	1.10–2.17	1.65 *	1.27–2.14
Arthritis	1.44 *	1.31–1.59	1.55 *	1.34–1.78	1.64 *	1.43–1.87
Depression	1.47 *	1.33–1.63	1.45 *	1.22–1.71	1.50 *	1.27–1.77
Kidney Disease	1.71 *	1.23–2.37	1.75 *	1.14–2.67	1.89 *	1.26–2.82
Diabetes	2.00 *	1.70–2.36	1.89 *	1.52–2.35	1.59 *	1.31–1.93
Overweight/obese	1.51 *	1.41–1.62	1.57 *	1.38–1.78	1.41 *	1.25–1.60
Current Smoker	1.11 *	1.01–1.23	1.75 *	1.50–2.04	1.94 *	1.68–2.62
Binge Drinking	0.94	0.87–1.01	1.26 *	1.10–1.44	1.14	0.99–1.30

* Significant Adjusted Odds Ratios.
